# A genetic association study of DNA methylation levels in the *DRD4* gene region finds associations with nearby SNPs

**DOI:** 10.1186/1744-9081-8-31

**Published:** 2012-06-12

**Authors:** Sophia J Docherty, Oliver SP Davis, Claire MA Haworth, Robert Plomin, Ursula D’Souza, Jonathan Mill

**Affiliations:** 1 King’s College London, MRC Social, Genetic and Developmental Psychiatry Centre,, Institute of Psychiatry, De Crespigny Park, Denmark Hill, London, SE5 8AF, UK

## Abstract

**Background:**

*Dopamine receptor D*_*4*_*(DRD4)* polymorphisms have been associated with a number of psychiatric disorders, but little is known about the mechanism of these associations. DNA methylation is linked to the regulation of gene expression and plays a vital role in normal cellular function, with abnormal DNA methylation patterns implicated in a range of disorders. Recent evidence suggests DNA methylation can be influenced by *cis*-acting DNA sequence variation, that is, DNA sequence variation located nearby on the same chromosome.

**Methods:**

To investigate the potential influence of *cis*-acting genetic elements within *DRD4*, we analysed *DRD4* promoter DNA methylation levels in the transformed lymphoblastoid cell-line DNA of 89 individuals (from 30 family-trios). Five SNPs located +/− 10kb of the promoter region were interrogated for associations with DNA methylation levels.

**Results:**

Four significant SNP associations were found with DNA methylation (rs3758653, rs752306, rs11246228 and rs936465). The associations of rs3758653 and rs936465 with DNA methylation were tested and nominally replicated (p-value < 0.05) in post-mortem brain tissue from an independent sample (N = 18). Interestingly, the DNA methylation patterns observed in post-mortem brain tissue were similar to those observed in transformed lymphoblastoid cell line DNA.

**Conclusions:**

The link reported between DNA sequence and DNA methylation offers a possible functional role to seemingly non-functional SNP associations. *DRD4* has been implicated in several psychiatric disease phenotypes and our results shed light upon the possible mode of action of SNP associations in this region.

## **Background**

*DRD4*, which encodes a G-protein-coupled dopamine receptor [1], has been widely implicated in the etiology of neuropsychiatric disease. Genetic associations have been reported between *DRD4* and ADHD [2-6], anorexia [7], schizophrenia [8,9], depression [10,11], obesity [12], addiction [13] and personality disorders [14]. Although some of these genetic associations are supported by evidence of altered *DRD4* expression in specific diseases [11,15,16], the mechanism by which *DRD4* polymorphisms influence behaviour remains unknown. Epigenetic functions may offer some explanation. Epigenetics refers to the reversible regulation of various genomic functions mediated through partially stable modifications of DNA and histone codes, excluding DNA sequence changes. Epigenetic processes, including histone modification and DNA methylation, are intrinsically connected to gene expression, allowing the regulation of gene function through non-mutagenic means [17]. The methylation of CpG dinucleotides, which are overrepresented in the promoter regions of many genes, acts to obstruct cells’ transcriptional machinery and silences gene expression. Correct control of DNA methylation is vital to normal cellular function, and DNA methylation dysfunction has been linked to a number of human pathologies [18,19], including complex neuropsychiatric phenotypes such as schizophrenia and bipolar disorder [20]. Though stochastic factors have been implicated [21], there is growing evidence for the importance of both environmental and genetic factors in the influence of DNA methylation.

Studies of twins suggest greater variability in the DNA methylation patterns of dizygotic (DZ) twins relative to monozygotic (MZ) twins, and heritability estimates of 0.20-0.97 have been generated for DNA methylation levels within various genomic regions [22,23]. A SNP in *MTHFR –* the gene encoding 5,10-methylenetetrahydrofolate reductase which is involved in the maintenance of DNA methylation patterns – has been linked to global DNA methylation levels [24,25]. Furthermore, several studies have demonstrated *cis*-acting genetic associations with DNA methylation in humans, chimpanzees and mice [20,26-34]. Crucially, as these genetic associations with DNA methylation levels have also been shown to correlate with levels of gene expression [33-36], they could represent the mechanism behind allele-specific gene expression, which has been commonly reported throughout the genome [37-40].

A function for previously unexplained genetic associations may therefore lie in the connection between DNA methylation and DNA sequence. If this is the case, further investigation of such markers might involve assessing their influence over local DNA methylation patterns. Yet several studies of DNA methylation report contradictory findings, indicating that the importance of genetic factors may vary across genomic regions, tissues and environments [23,41,42]. Thorough analysis in relevant tissues is therefore necessary to draw conclusions about any one gene of interest.

In the present study, we assess the potential influence of *cis*-acting genetic polymorphisms in mediating DNA methylation at 9 CpG sites across the *DRD4* promoter region, using lymphoblastoid cell-lines from a familial sample, and post-mortem brain tissue from an independent set of individuals.

## **Methods**

### Samples

#### *CEPH*

We obtained 89 high-quality Centre d'Etude du Polymorphisme Humain (CEPH) genomic DNA samples extracted from transformed lymphoblastoid cell lines (Coriell Institute for Medical Research, NJ, USA). All samples were tested for degradation and quantified in triplicate using fluorimetry, employing PicoGreen® dsDNA quantitation reagent (Cambridge Bioscience, UK). Aliquots of each sample were diluted 1:5 with TE buffer (10 mM Tris, 1 mM EDTA) to a working concentration of 50 ng/μl.

### *Brain samples*

Post-Mortem brain samples were obtained from the 18 individuals archived in the MRC London Brainbank for Neurodegenrative Disease (Maudsley Brain Bank, Department of Neuropathology, Institute of Psychiatry, London, UK). Brain tissue samples from normal (N = 7; 2 females and 5 males) and Alzheimer’s patients (N = 11; 6 females and 5 males) were used, with agonal states including: Bronchopneumonia, cardiac failure, hypertension, Pulmonary Embolus, Sideroblastic anaemia, coronary occlusion, carcinoma of left kidney, Myocardial Infarction and Ischemic heart disease. Tissue was obtained from multiple brain regions from each patient: striatum, cerebellum, mid-brain, superior frontal gyrus (SFG) and superior temporal gyrus (STG). Data from the Allen Brain Atlas showed *DRD4* to be expressed in these tissues (Allen Brain Atlas Resources [Internet]. Seattle (WA): Allen Institute for Brain Science. ©2009. Available from: http://www.brain-map.org). Tissue samples were between 0.5-1g and stored at −70°C, prior to use. Total DNA was prepared from homogenized tissue using the Qiagen Allprep DNA mini kit.

### DNA methylation analysis

Bisulfite-PCR primers spanning a region in the *DRD4* promoter were designed using the online Sequenom EpiDesigner software ( http://www.epidesigner.com). Sodium bisulfite treatment was performed on 375 ng of each CEPH sample using the EZ-96 DNA Methylation Kit (Zymo Research, CA, USA) following the manufacturers’ standard protocol. The DNA of all 89 individuals was bisulfite converted in the same 96-well plate, with fully-methylated (a methylation level of 100%) and unmethylated DNA (0% methylation) samples included as assay controls. Bisulfite-PCR amplification was conducted using Hot Star *Taq* DNA polymerase (Qiagen, UK) and cycling conditions of 45 cycles with an annealing temperature of 56°C. The primers (F: GGGATTTTTTGTTTAGGGTTAGAGG, R: CACCCTAATCCACCTAATATCTAACA) amplified the region chr11:626,509-626,904, assessing 19CpG units (32 CpG sites). DNA methylation analysis was conducted using the Mass-spectrometry-based Sequenom EpiTYPER system (Sequenom Inc, CA, USA), as described previously [43,44]. The entire experiment, from sodium bisulfite treatment onwards, was subsequently repeated in duplicate to control for technical variation.

To optimise reliability, the data produced were subject to a number of quality control measures. CpG site-containing fragments with a mass outside the range measurable by the Sequenom Epi-Typer system were excluded from further analyses. CpG site-containing fragments with equal or overlapping masses – making them irresolvable by mass spectrometry – were also excluded. CpG site-containing fragments whose measurement was potentially confounded by single nucleotide polymorphisms according to dbSNP build 130 were also discarded. This included fragments containing SNPs which had either been shown to effect populations of European ancestry, or had not yet been tested in such populations. Fragments whose enzyme cut-sites could be affected by SNPs were also excluded. As bisulfite treatment converts cytosines to thymines, fragments containing a C/T SNP on the assayed strand were not excluded, unless this SNP was within a CpG position. Finally, CpG site-containing fragments with >33.33% missing data were excluded. Additional file 1 details the exclusions. To minimize technical variability, the remaining methylation data were averaged across the two replicates (the average correlation between the first and second round DNA methylation levels across the nine CpG units was 0.81). All subjects had < 25% missing DNA methylation data. The mean of the DNA methylation values for all CpG sites was calculated in order to gauge the average DNA methylation level in each subject.

### SNP Genotyping

**Figure 1:**
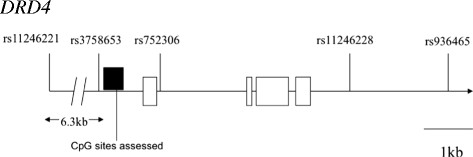
**Positional overview of *DRD4 *region investigated for SNP associations with DNA methylation.** White boxes = exons; Black box = region assessed for DNA methylation. Diagram created using data from UCSC ( http://genome.ucsc.edu/) and HapMap ( http://hapmap.ncbi.nlm.nih.gov/).

SNP genotypes for the extensively investigated CEPH sample (see [45] for further description) were downloaded from the HapMap website on April 8th 2009 ( http://hapmap.ncbi.nlm.nih.gov/ - HapMap Rel 27 Phase II + III, Feb 09, NCBI 36 assembly, dbSNP b126). Genotypes for SNPs within 10kb up- and downstream of the DNA methylation-assayed region were downloaded. 6 SNPs with an MAF <0.05 were excluded from the analysis. The remaining SNP genotypes for the 89 individuals within our sample were input into Haploview, where the ‘tagger’ function was used, with an r^2^ threshold of 0.8, to select 5 SNPs offering optimum coverage (rs11246221, rs3758653, rs752306, rs11246228, rs936465) of the region [46]. All SNPs were in Hardy-Weinberg equilibrium at the p > 0.01 level. Figure 1 graphically depicts the *DRD4* gene region investigated. 

The significant associations of two SNPs – rs3758653 and rs936465 – were tested for replication in the 18 Maudsley Brain Bank samples. The SNPs were genotyped via restriction enzyme digestion of PCR products, followed by size descrimination using gel electrophoresis. The primers CCCTCCACTCCAGGCCTCCC and CCTCCATTCCCTCCGGCCCA were used to amplify a 347bp region surrounding rs3758653, which was then digested using EcoRI according to the manufacturer’s instructions. EcoRI cuts the PCR product only if the C allele is present. The primers GCTGCCACCCTACCCCAGGT and GCACTGGGCTGGGCCTGAAC were used to amplify a 203bp region surrounding rs936465, which was then digested with Hpy166II according to the manufacturer’s guidelines. Hpy166II selectively cuts the PCR product if the G allele is present on the genomic + strand. Both SNPs had an MAF > 0.05 and were in Hardy-Weinberg equilibrium at the p > 0.01 level.

### Statistical Analyses

The Sequenom Epityper system measures DNA methylation levels as a proportion: 0 (unmethylated) to 1 (fully-methylated). The DNA methylation data was therefore bounded by 0 and 1, and CpG sites with average methylation levels close to these boundaries had a truncated variance. To overcome the problem of skewed variance, the DNA methylation data were arcsine transformed. The data were then normalised using a Van der Waerden transformation and standardized to a mean of 0 and standard deviation of 1. Sex was not associated with *DRD4* DNA methylation and so was not controlled for in our analyses. Each SNP was tested for association with the average DNA methylation level across all CpGs measured in the CEPH sample, using linear mixed effects models in R [47]. SNP genotype was entered as a fixed effect into the model. As the sample consisted of trios, the genetic relatedness between mother and offspring, and father and offspring, was controlled for as a random effect in the model. We were also able to control for the environment shared by each nuclear family. Unfortunately, our sample size was too small to accurately test for population stratification, however, as our sample consisted of CEPH individuals of European ancestry, and as all SNPs were in Hardy-Weinberg equilibrium, we would not expect significant population stratification. As the SNPs were correlated, we used Li and Ji’s method to estimate the effective number of SNPs tested (MeffLi [48]; calculated at http://gump.qimr.edu.au/general/daleN/SNPSpD/[49]), followed by the Bonferroni method to correct for multiple testing. SNPs exhibiting an association with overall DNA methylation levels were then tested for association with each individual CpG unit within a region. Effect sizes were estimated from Ns and Chi^2^ values using an online Effect Size Calculator ( http://myweb.polyu.edu.hk/~mspaul/calculator/calculator.html).

For the post-mortem brain samples, the *DRD4* DNA methylation data were transformed and standardized using the methods described above. Sex and Alzheimer’s status were not associated with *DRD4* DNA methylation and so were not controlled for in our analyses. rs3758653 and rs936465, the two strongest associations, were tested for association with average DNA methylation across all CpGs in each available brain tissue type using linear regression in R. Nominally significant associations were investigated further by testing the SNP’s association with individual CpG sites.

### Power

Power was estimated using the Genetic Power Calculator [50]. Under the additive association model used, at the p < 0.05 level our sample of 89 individuals from 30 CEPH trios had 80% power to detect a causal variant of 20% allele frequency and 9.6% effect size; and a marker in linkage disequilibrium (D’ = 0.8) with a causal variant of 20% allele frequency and 15.1% effect size. Our sample of 18 post-mortem brain samples had 80% power to detect a causal variant of 20% allele frequency and 41.5% effect size; and a marker in linkage disequilibrium (D’ = 0.8) with a causal variant of 20% allele frequency and 64.8% effect size.

### *eQTL assessment*

SNPs demonstrating significant associations with DNA methylation in the CEPH sample were assessed for eQTL status using SCAN ( http://www.scandb.org) [51]. This online resource contains association p-values between genotype and expression data generated from the transformed lymphoblastoid cell lines of CEPH and YRI (Yoruba subjects from Ibadan, Nigeria) subjects included in the HapMap project [45].

## **Results**

### SNP associations with average DNA methylation levels

**Figure 2:**
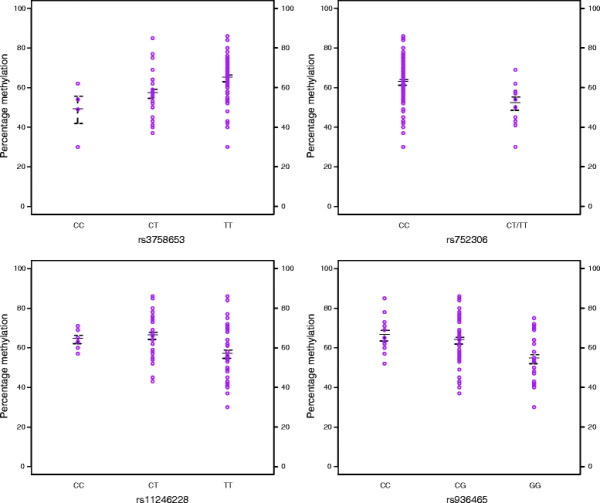
**Significant SNP associations with average DNA methylation levels in the *DRD4 *gene region.** Average percentage DNA methylation plotted by genotype at associated SNPs. Thick black lines represent group mean. Whiskers mark one standard deviation from the mean. As our sample contained only one TT homozygote at rs752306, we collapsed it into the heterozygote groups for analysis.

Figure 1 graphically depicts the gene region investigated in this study. The mean DNA methylation level across this region within our CEPH transformed lymphoblastoid cell line sample was 0.61, and the standard deviation was 0.13. Table 1 displays the results of SNP association analyses of the average *DRD4* DNA methylation level. The relatedness of the CEPH sample was controlled for in the analyses. Mean DNA methylation values across all CpGs were used, however, the first principal component generated from principal components analysis produced similar results (results not shown). Significant SNP associations are represented in Figure 2, with average DNA methylation plotted against genotype. 4 out of 5 investigated SNPs showed significant associations with average DNA methylation (rs3758653: N = 81; d.f. = 1; Chi^2^ = 12.02; P-value = 0.001, rs752306: N = 81; d.f. = 1; Chi^2^ = 6.78; P-value = 0.009; rs11246228: N = 81; d.f. = 1; Chi^2^ = 8.67; P-value = 0.003, rs936465: N = 89; d.f. = 1; Chi^2^ = 10.61; P-value = 0.001). The effect sizes of these associations were substantial – with rs3758653, rs752306, rs11246228 and rs936465 respectively accounting for 14.8%, 8.4%, 10.7% and 11.9% of the variance in average DNA methylation across the *DRD4* region in our sample – although the absolute differences in DNA methylation values across genotype groups were small to modest. Although the ranges of DNA methylation shown in Figure 2 are distinct for opposing homozygote groups, both groups overlap with the heterozygote group at all SNPs. The associations remained significant after Bonferroni correction for the 4 effective SNP tests conducted (MeffLi) [48]. As the sample contained only one TT homozygote at rs752306 the TT and CT groups were collapsed for the association analyses. When the true genotypes were used the association remained nominally significant (N = 81; d.f. = 1; Chi^2^ = 9.45; P-value = 0.002). 

**Table 1:** **SNP associations with average DNA methylation levels in the *DRD4 *promoter region**

**Gene region**	**DNA Methylation assessed region**	**SNP**	**Position**	**N**	**Allele A**	**Allele B**	**Allele A Freq**	**Mean AA**	**Mean AB**	**Mean BB**	**Chi**	**P-value**
*DRD4*	chr11:626,509-626,904	rs11246221	620124	81	A	G	0.69	0.62	0.62	0.52	1.79	0.181
		rs3758653	626399	81	C	T	0.22	0.49	0.57	0.65	12.02	0.001
		rs752306	627622	81	C	T	0.93	0.63	0.52	-	6.78	0.009
		rs11246228	631563	81	C	T	0.28	0.64	0.66	0.57	8.67	0.003
		rs936465	633568	89	C	G	0.42	0.66	0.64	0.54	10.61	0.001

**Figure 3:**
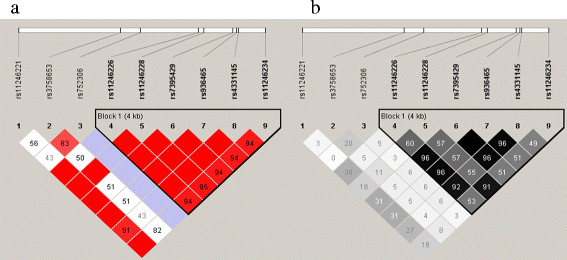
**LD pattern (a = D’, b = r**^**2 **^**) across 9 SNPs associated with average DNA methylation in the *DRD4 *gene region.**

Figure 3 displays the LD pattern between the 5 *DRD4* SNPs tested in our sample. The four extra SNPs whose associations were captured (according to the Haploview ‘tagger’ function at r^2^ > 0.8) are also included in Figure 3. The high LD shown between rs11246226, rs11246228, rs7395429, rs936465, rs4331145 and rs11246234 suggests that the significant associations of rs11246228 and rs936465 (and by proxy, the four other SNPs) with DNA methylation are likely to reflect the same effect. As the CEPH population has been extensively genotyped, one of these SNPs could be the influential variant. As rs3758653 and rs752306 exhibit generally weaker LD relationships, they may reflect independent effects. When the effects of rs3758653 and rs936465 are considered together, the association with average *DRD4* DNA methylation is stronger (N = 81; d.f. = 2; Chi^2^ = 16.38; P-value = 2.79E-04), further suggesting the effects of rs3758653 and rs936465 may be independent. As might be expected from Figure 3, adding rs752306 and rs11246228 does not further increase the strength of the association (N = 81; d.f = 4; Chi = 18.79; P-value = 0.001). Similarly, haplotype analyses of rs3758653, rs752306, rs11246228 and rs936465 in UNPHASED [24] also indicated independence in the effects of rs3758653 and rs936465, with rs752306 and rs11246228 exerting no extra effects (data not shown). 

As one might expect SNPs associated with DNA methylation to also show associations with gene expression, the four significant *cis* SNP associations were investigated further using the online tool SCAN, which contains p-values from association tests of genotype and expression data from the transformed lymphoblastoid cell lines of CEPH and YRI HaPMaP subjects [51]. No evidence for *cis* effects on gene expression was found. As SCAN only returns information on genetic associations with a significance levels of p < 0.0001, it is possible that weaker associations were present, however, the lack of strong associations with gene expression raises questions about the function of the SNP associations identified.

Many CpG sites were removed from this study during the stringent quality control process. Some of the CpG units excluded from the analysis of the *DRD4* region are of interest because the existence of CpG sites depends on SNP genotypes. CpG site 29 on CpG unit 28.29 may be affected in this way by rs12720379 genotype. rs3758653 and rs752306 showed significant associations with DNA methylation at CpG unit 28.29. In most cases the correlations of CpG unit 28.29 with those showing strong SNP associations in the initial analyses of the *DRD4* region are significant and modest (0.23-0.41). Though rs12720379 is an unvalidated SNP which has yet to be tested in a sample of European ancestry, its influence over the presence of a CpG site, and therefore the presence of DNA methylation, can not be ruled out. Neither, therefore, can its possible role in the significant SNP associations reported here.

### SNP associations with individual CpG units in the *DRD4* gene region

**Figure 4:**
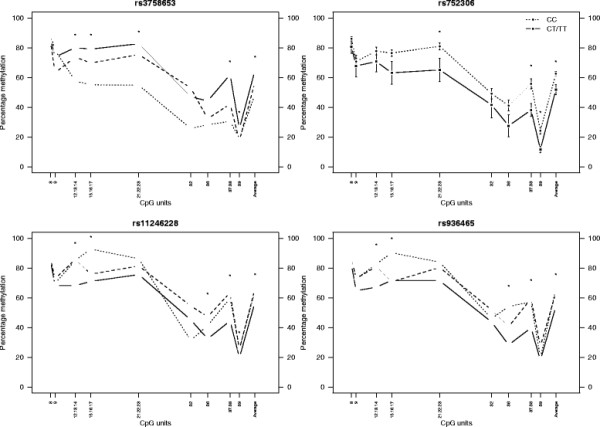
**Average percentage DNA methylation across CpG sites in the *DRD4 *region by genotype groups at 4 associated SNPs.** Error bars denote standard deviation. * = significant associations at the p < 0.05 level. Though association analyses were conducted on arcsine transformed data, the true proportion of methylation observed is plotted here. The one homozygous TT subject at rs752306 within our sample was collapsed with the heterozygote group in association analyses.

**Figure 5:**
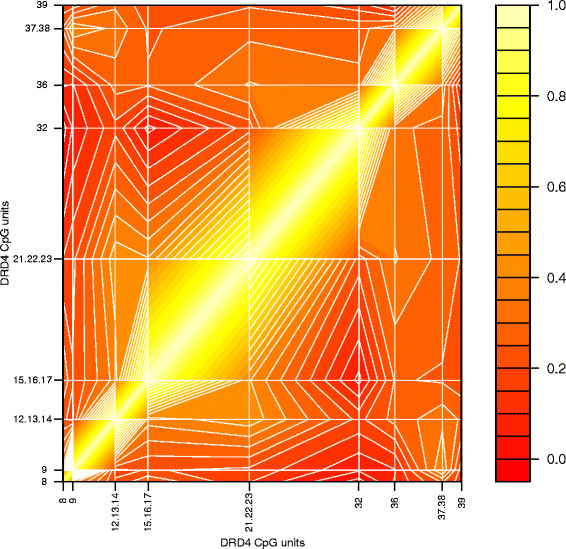
**Correlation across CpG sites within the *DRD4 *region.** Colours represent Pearson’s product–moment correlations between DNA methylation levels at DRD4-association CpG sites. Colour key is provided to the right of the plot.

We explored the 4 significant SNP associations with average *DRD4* methylation levels further. Table 2 contains p-values from the association analyses of rs3758653, rs752306, rs11246228 and rs936465 with DNA methylation at individual *DRD4* CpG units, and Figure 4 plots average DNA methylation levels by genotype group at the 4 associated SNPs. The EpiTyper system was unable to resolve all CpG sites, and so while some of the measured CpG units refer to a single CpG site, many reflect average levels of DNA methylation across multiple CpG sites. The similar patterns of association seen between rs11246228 and rs936465 reflect the modest LD between these two SNPs shown in Figure 3. The significant associations of rs11246228 and rs936465, as well as of rs3758653, with CpG units 12.13.14, 15.16.17, 21.22.23, 36 and 37.38 reflect the modest correlations between DNA methylation levels across these sites – shown in Figure 5’s heatmap displaying the similarities in DNA methylation levels across the measured *DRD4* region.

**Table 2:** **SNP associations with DNA methylation at CpGs within the *DRD4 *gene region**

		** P-values**			
CpG unit	bp position	rs3758653	rs752306	rs11246228	rs936465
8	@107	0.379	0.212	0.510	0.082
9	@113	0.135	0.791	0.225	0.083
12.13.14	@139, 149 & 151	0.027	0.248	0.001	0.007
15.16.17	@159, 168 & 170	0.007	0.075	0.015	0.012
21.22.23	@221, 224 & 227	0.007	0.039	0.125	0.077
32	@288	0.652	0.358	0.486	0.418
36	@310	0.148	0.184	0.027	0.006
37.38	@339, 346	0.000	0.047	0.002	0.011
39	@351	0.019	0.005	0.076	0.295

### *Association of rs3758653 and rs936465 with DRD4DNA methylation in post-mortem brain samples from five brain regions*

*DRD4* is known to be expressed widely in the brain [52](Allen Brain Atlas Resources [Internet]. Seattle (WA): Allen Institute for Brain Science. ©2009. Available from: http://www.brain-map.org). As they appeared to represent independent effects, we tested the association of rs3758653 and rs936465 with *DRD4* DNA methylation for replication within a sample of post-mortem brain tissue from 5 brain regions: striatum, mid-brain, cerebellum, superior temporal gyrus (STG) and superior frontal gyrus (SFG). Average levels of DNA methylation in the *DRD4* region in our sample were as follows: Striatum mean = 0.52, s.d = 0.06; Mid-brain mean = 0.48, s.d = 0.05; Cerebellum mean = 0.52, s.d = 0.06, STG mean = 0.54, s.d = 0.03, SFG mean = 0.56, s.d = 0.06. The SNP-association results are given in Table 3. One-tailed p-values are provided as we expected effects in the same direction as was observed in the CEPH transformed lymphoblastoid cell line DNA. Of the 10 association results, 8 followed the same direction of effect as the original SNP associations. Nominally significant associations (p < 0.05) were found between rs3758653 and SFG DNA methylation (N = 9; d.f. = 1 and 7; F = 6.51; r^2^ = 0.45; P-value = 0.017), and between rs936465 and Mid-brain (N = 9; d.f. = 1 and 7; F = 6.51; r^2^ = 0.44; P-value = 0.017) and STG DNA methylation (N = 12; d.f. = 1 and 10; F = 6.31; r^2^ = 0.39; P-value = 0.015). Again, the effect sizes were large, with the SNPs accounting for 39-45% of the variation in DNA methylation (rs3758653: Allele A=C, Allele B =T; rs936465: Allele A=C, Allele B = G. STG= Singular Temporal Gyrus; SFG = Singular Frontal Gyrus); however the absolute differences in DNA methylation were small to moderate, the sample size was small and the associations did not remain significant after Bonferroni correction for the 10 tests conducted. Although analyses utilized transformed data, the true DNA methylation levels are given here. Where only one homozygote was available, this subject was combined with the heterozygotes for analyses. The one rs3758653 heterozygote with SFG methylation data was combined with the TT homozygote group for analyses. One-tailed p-values are provided. 

**Table 3:** **Associations of rs3758653 and rs936465 with average *DRD4 *DNA methylation in post-mortem brain tissue**

**SNP**	**Tissue**	**N**	**Mean AA (N)**	**Mean AB (N)**	**Mean BB (N)**	**F**	**P-value**
rs3758653	Striatum	13	0.47 (2)	0.57 (3)	0.51 (8)	0.03	0.433
	Mid-brain	8	0.38 (1)	0.53 (2)	0.48 (5)	0.06	0.590
	Cerebellum	9	0.51 (2)	NA (0)	0.53 (7)	0.10	0.382
	STG	12	0.52 (1)	0.57 (3)	0.52 (8)	3.05	0.944
	SFG	9	0.48 (2)	0.59 (1)	0.57 (7)	6.51	0.017
rs936465	Striatum	13	0.56 (3)	0.5 (7)	0.53 (3)	0.09	0.384
	Mid-brain	8	0.52 (3)	0.46 (4)	0.42 (1)	5.82	0.038
	Cerebellum	9	0.56 (3)	0.51 (3)	0.5 (3)	3.02	0.063
	STG	12	0.55 (5)	0.53 (5)	0.52 (2)	6.31	0.015
	SFG	10	0.6 (3)	0.54 (4)	0.53 (3)	2.93	0.063

**Figure 6:**
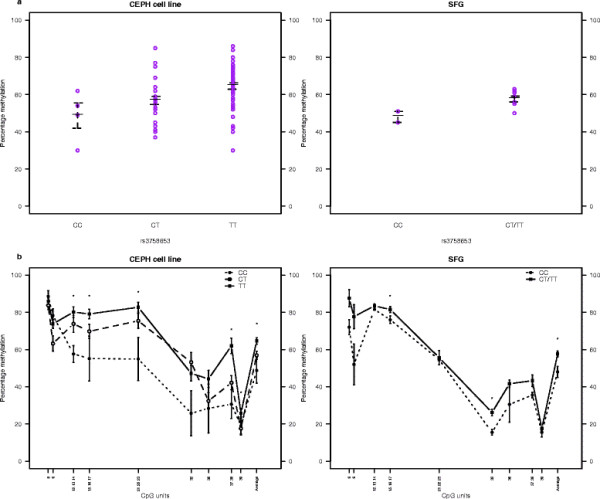
***DRD4 *percentage DNA methylation levels in CEPH transformed lymphoblastoid cell lines and post-mortem Singular Frontal Gyrus tissue (SFG). a**) Average *DRD4* DNA methylation levels plotted by rs3802971 genotype **b**) DNA methylation levels across individual *DRD4* CpG sites plotted by rs3802971 genotype group. * = significant associations at the p < 0.05 level. Though arcsine transformed data were used in analyses, the true proportion of DNA methylation observed is plotted here. The one heterozygous subject in the post-mortem brain tissue sample was combined with the TT homozygotes for analyses.

**Figure 7:**
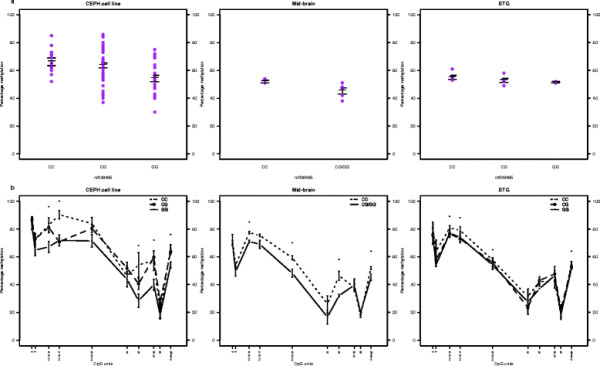
***DRD4 *percentage DNA methylation levels in CEPH transformed lymphoblastoid cell lines and post-mortem Mid-brain and Singular Temporal Gyrus tissue (STG). a**) Average *DRD4* DNA methylation levels plotted by rs936465 genotype **b**) DNA methylation levels across individual *DRD4* CpG sites plotted by rs936465 genotype group. * = significant associations at the p < 0.05 level. Though arcsine transformed data were used in analyses, the true proportion of DNA methylation observed is plotted here. The one subject with available mid-brain DNA methylation data who was GG homozygous at rs936465 was combined with the heterozygotes for analyses.

Figures 6 and 7 display the nominally significant associations of rs3758653 and rs936465 with average *DRD4* DNA methylation in post-mortem brain tissue, as well as the associations of these SNPs with individual CpG sites within the *DRD4* region. Comparison with data from the CEPH sample indicates not only a similar pattern of association results, but also a similar pattern of DNA methylation levels across the *DRD4* CpG sites.

## **Discusion**

Our investigation uncovered significant SNP associations with DNA methylation levels in the *DRD4* promoter, which were replicated at a nominal level of significance (p < 0.05) in an independent sample of post-mortem brain tissue. As with the majority of genetic effects identified in previous studies, these SNP associations occurred in *cis*[20,26-32]. As *cis*-acting genetic influence over DNA methylation has been observed throughout the genome, it may account for many previously unexplained genetic associations. Though we did not analyse *trans-*acting SNPs in the present study, *trans* genetic effects are also likely to be important [26,35].

The greatest group difference in DNA methylation was 16%, between the two rs3758653 homozygote groups. At this stage in our understanding, the functional relevance of these small changes in DNA methylation is unknown. If one is attempting to find an influential locus of large effect, substantial changes in DNA methylation levels may be expected. However, as most complex phenotypes are now thought to be influenced by a myriad of factors of small effect [53,54], more subtle differences in DNA methylation levels may be important. Our knowledge is still very limited, but as a ~20% difference in DNA methylation has been previously shown to associate with a 2-fold change in gene expression [35,55], or even to bring about the presence or complete absence of gene expression across various tissues [56], it is likely that individual differences in phenotypic outcome will be cumulatively influenced by many small differences in the epigenetic, and consequently the transcriptomic, landscape. Detecting genetic influences over even more modest individual differences in DNA methylation than observed here will require far larger samples.

It is likely that *cis*-acting DNA effects on DNA methylation are more important in some genomic regions than in others [22,26,33,35], and our findings suggest that *DRD4* may represent a region in which *cis-*acting genetic-control commonly occurs. Although previous genomewide association studies of DNA methylation have not reported positive results from the *DRD4* region [10,17,19], such investigations may be limited in the CpGs and SNPs they were able to investigate by the laboratory platforms used. The *DRD4* SNPs tested did not show *cis*-associations with expression in the SCAN database [51], and although we found *DRD4* SNP associations with DNA methylation in our independent replication sample, they did not withstand Bonferroni correction for multiple testing. Furthermore, the findings of a recent twin study of the same *DRD4*-associated region assayed here are inconsistent with those we have reported. Wong et al.’s investigation of DNA methylation in 46 MZ and 45 DZ twins indicated no heritable element was involved [42]. Differences in the sample and tissue types used across the two studies are likely to explain the disparate findings. Additionally, all investigations of this region to date – including the present study – have involved extremely small sample sizes. Future work will require far larger samples in order to draw firm conclusions.

One limitation of this study was the modest size of both the discovery and replication samples. Our discovery sample had 80% power to detect causal QTLs of 9.6% effect size, or markers in linkage disequilibrium (D’ = 0.8) with causal QTLs of 15.1% effect size. As subjects were drawn from the extensively characterized CEPH sample, we were more likely to have access to the genotypes of causal variants. Furthermore, one might expect *cis*-acting SNPs to show larger effects over local DNA methylation than over complex disease phenotypes. Nonetheless, as DNA methylation levels are likely be subject to the effects of multiple environmental, *cis* and *trans* genetic, and also stochastic factors, far smaller effect sizes may be involved than the sample was equipped to detect. Though we excluded SNPs with MAFs below 5%, the MAF of one of the SNPs associated with DNA methylation was lower than 10% (rs752306 - see Table 1), further stretching the power of our sample.

Our replication sample was also limited in size, with the analyses involving the largest N of 13 having only 80% power to detect a causal QTL of 53% effect size. Though we did detect nominal SNP associations, we feel our replication sample was too small to draw final conclusions. Unfortunately, the laboratory techniques used to assess DNA methylation are relatively new and still rapidly developing, and both genetically and epigenetically assessing samples involves considerable cost and labour. Consequently, to date the investigations of genetic influences over DNA methylation have all involved similarly small sample sizes [20,22,26,30,33,35,42]. Wisdom gained from studies of other complex phenotypes dictates that future studies should aim to include far larger sample sizes if they hope to detect the expected small effects [53,57,58]. This wisdom can also be applied when interpreting the relatively large effect sizes (8.4-14.8%) we did manage to detect in our small discovery sample, and the even larger effect sizes (39-44%) we observed in our replication sample. Though many significant associations between candidate genes and complex traits have been identified and replicated over the years, the large effect sizes originally reported in discovery samples often fall as sample sizes, and number of replication studies, increase [3]. We would therefore expect the effects found in any future investigations of larger samples to be smaller than those reported here.

Our small sample size also restricted the statistical analyses we were able to perform. Firstly, although we would not expect to find significant population stratification within our CEPH participants, the small sample size did not permit us to test this empirically. Secondly, though we controlled for the effects of genetic relatedness and nuclear family environment in our association analyses, our sample was too small to simultaneously estimate many variance parameters accurately. As a result, in many cases the effect of the family environment could not be reliably distinguished from the effects of genetic relatedness in our sample. Although the influence of the environment over DNA methylation is well documented [59], we predicted that it would be less significant in the transformed lymphoblastoid cell line DNA used here. Indeed, after controlling for genetic relatedness in our analyses, the family environment often showed no additional influence over DNA methylation. Our modest sample size also left us unable to take parent of origin effects into account, which recent computational analyses suggest are prevalent across the genome [60]. The stringent Bonferroni method used for multiple-testing correction in our replication sample may also be seen as a limitation, as the 5 tissues tested came from largely the same participants. However, DNA methylation across the 5 brain regions within individuals was uncorrelated, likely to be in part due to low levels of variation in our sample.

The quantitative DNA methylation data generated using the MALDI-TOF-based Sequenom EpiTyper technique were limited in a number of ways. As Additional File 1 demonstrates, due to the position of cut sites, after base-specific RNA cleavage some adjacent CpGs remained on the same fragment [33,34]. As a result, many of the CpG units assessed – including some of those exhibiting significant SNP associations – consisted of average DNA methylation measurements across several CpG sites. Additional File 1 also highlights the exclusion of numerous CpG-containing fragments for a variety of reasons. Since the present study was conducted, R packages such as RSeqMeth [44] and MassArray [61] have been created to assist researchers in designing assays which avoid at least some of this loss of data. Furthermore, an optimal method for examining associations between genetic markers and DNA methylation would examine allele-specific methylation The interpretation of results from future studies might be aided by an approach which uses resources such as SCAN to select known eQTLs for tests of association with DNA methylation levels [51].

The source of the DNA used in our discovery sample represents another possible limitation. The aim of this study was to assess the influence of DNA sequence over DNA methylation in 5 genomic regions. By assessing DNA methylation in the CEPH sample, we had the opportunity to investigate a large number of SNPs at no extra cost to our laboratory. However, comparisons of RNA extracted from transformed lymphoblastoid cell lines to that extracted directly from blood cells have revealed significant differences in gene expression [62]. Moreover, when DNA methylation in lymphoblastoid cell lines from type 1 diabetes patients was compared with that in paired peripheral blood leucocytes, differences were observed in 8% of the genes assessed [63]. The SNP associations identified here may therefore not apply to *in vivo* DNA methylation levels. Despite this, we nominally replicated the two SNP associations emerging from the analysis of the CEPH sample, in DNA derived from brain tissue. It is also worth noting that Figures 6 and 7 indicate very similar patterns of *DRD4* DNA methylation in the cell line and brain-tissue derived DNA analysed here. Furthermore, a 2010 study of the exact *DRD4* region studied here found similar levels of methylation in DNA extracted from buccal swabs [42], suggesting that the results of DNA methylation analyses in transformed lymphoblastoid cell lines may be relevant *in vivo*.

Future investigations will benefit from an approach similar to that used in Schalkwyk et al.’s 2010 study, which assesses the effects of different alleles within the same individual [35,64]. This enables a test of SNP association against a background controlled entirely for all environmental and other genetic factors, and unlike our approach, in heterozygotes at least it can expressly identify DNA methylation differences across the two separate DNA strands. As none of the SNPs identified in our study are known to effect the expression of proximal genes, it is difficult to draw conclusions regarding the effect of the associations with DNA methylation that we have observed. The interpretation of results from future studies might be aided by an approach which uses resources such as SCAN to select known eQTLs for tests of association with DNA methylation levels [51].

Although the limitations of transformed lymphoblastoid cell line DNA have been discussed above, and though we did not consider disease phenotypes in our analyses, our findings may have implications for research into *DRD4 *disease-associations, especially given the nominally significant associations we found in post-mortem brain tissue. *DRD4* has been previously linked to a number of psychiatric and behavioural disorders, most notably ADHD [2]. Much of the emphasis has been upon the exon 3 VNTR [3], yet SNPs in the *DRD4* promoter have also shown significant associations with ADHD [4,5], schizophrenia [8,9] and fibromyalgia [65]. Interestingly, two SNPs tagged in this study have emerged previously in the literature (see Figure 3); rs936465 is in LD (r2 of 0.96) with rs4331145, which has been implicated in schizophrenia [9], and rs11246226 (r2 of 0.55), which has been implicated in schizophrenia and fibromyalgia [9,65]. DNA methylation has been suggested as a mediator of well-known environmental influences over disease phenotypes such as ADHD [59,66,67]. Our results suggest that epigenetic processes may mediate previously identified, but as yet unexplained, genetic influences too.

## **Conclusions**

We have reported SNP associations with DNA methylation levels in the *DRD4* gene region. Although replication is needed in larger samples, our findings add to the existing literature linking genetic sequence to DNA methylation patterns. *DRD4* has been implicated in a number of disease phenotypes, and our results offer a possible mechanism of action for previously unexplained SNP associations in these regions.

## **Competing interests**

The authors declare no competing interests.

## **Authors’ Contributions**

SJD generated DNA methylation data, genotyped the replication sample, ran statistical analyses and drafted the manuscript. OD and CH assisted in the conception of the project and in statistical analyses. RP, JM and UD assisted in the conception of the project and in drafting the manuscript. All authors read and approved the final manuscript.

## Supplementary Material

Additional file 1 CpG sites: assayed and excluded. Click here for file
