# Supplementary material for: A genetic association study of DNA methylation levels in the DRD4 gene region finds associations with nearby SNPs
Source: Behav Brain Funct. 2012 Jun 12;8:31. doi: 10.1186/1744-9081-8-31 (PMC3538530; doi:10.1186/1744-9081-8-31)
Supplement: Additional file 1 — CpG sites: assayed and excluded. [file 1744-9081-8-31-S1.doc]

Additional File 1: CpG sites: assayed and excluded

| Locus_CpG-unit | CpG sites | Reason for removal |
| --- | --- | --- |
| DRD4_1 | CpG 1 | Adjacent to rs12720369 |
| DRD4_2.3 | CpGs 2 & 3 | Adjacent to rs12720370, rs11246223 and rs12720370 |
| DRD4_4.5 | CpGs 4 & 5 | Mass overlap with unit 14 |
| DRD4_6 | CpG 6 | Adjacent to rs12720371, rs11246224 and rs12720371 |
| DRD4_7 | CpG 7 | Adjacent to rs12720372 |
| DRD4_8 | CpG 8 |  |
| DRD4_9 | CpG 9 |  |
| DRD4_10.11 | CpGs 10 & 11 | >33.33% missing data |
| DRD4_12.13.14 | CpGs 12 & 13 & 14 |  |
| DRD4_15.16.17 | CpGs 15 & 16 & 17 |  |
| DRD4_18.19.20 | CpGs 18 & 19 & 20 | High mass and adjacent rs12720375, rs67792664, rs747303,  rs2396543, rs12720377, rs10902180, rs12720378 and rs3842250 |
| DRD4_21.22.23 | CpGs 21 & 22 & 23 |  |
| DRD4_24.25.26 | CpGs 24 & 25 & 26 | Mass overlap with unit 20 |
| DRD4_27 | CpG 27 | Mass overlap with unit 3 |
| DRD4_28.29 | CpGs 28 & 29 | Adjacent to rs12720379 |
| DRD4_30 | CpG 30 | Adjacent to rs12720380 |
| DRD4_31 | CpG 31 | Low mass |
| DRD4_32 | CpG 32 |  |
| DRD4_33 | CpG 33 | >33.33% missing data |
| DRD4_34.35 | CpGs 34 & 35 | Mass overlap with unit 13 |
| DRD4_36 | CpG 36 |  |
| DRD4_37.38 | CpGs 37 & 38 |  |
| DRD4_39 | CpG 39 |  |

***Note:* Some CpG units cover multiple CpG sites.**
